# Chronic Wasting Disease Transmission Risk Assessment for Farmed Cervids in Minnesota and Wisconsin

**DOI:** 10.3390/v13081586

**Published:** 2021-08-11

**Authors:** James M. Kincheloe, Amy R. Horn-Delzer, Dennis N. Makau, Scott J. Wells

**Affiliations:** 1 Center for Science in the Public Interest, 1220 L St. N.W., Suite 300, Washington, DC 20005, USA; 2 Department of Veterinary Population Medicine, University of Minnesota, 225 Vet Med Ctr, 1365 Gortner Avenue, St. Paul, MN 55108, USA; dmakau@umn.edu (D.N.M.); wells023@umn.edu (S.J.W.); 3 Wisconsin Department of Agriculture, Trade, and Consumer Protection, 2811 Agriculture Drive, Madison, WI 53708, USA; Amy.HornDelzer@wisconsin.gov

**Keywords:** chronic wasting disease, transmissible spongiform encephalopathy, transmission, cervid, prion, risk analysis

## Abstract

CWD (chronic wasting disease) has emerged as one of the most important diseases of cervids and continues to adversely affect farmed and wild cervid populations, despite control and preventive measures. This study aims to use the current scientific understanding of CWD transmission and knowledge of farmed cervid operations to conduct a qualitative risk assessment for CWD transmission to cervid farms and, applying this risk assessment, systematically describe the CWD transmission risks experienced by CWD-positive farmed cervid operations in Minnesota and Wisconsin. A systematic review of literature related to CWD transmission informed our criteria to stratify CWD transmission risks to cervid operations into high-risk low uncertainty, moderate-risk high uncertainty, and negligible-risk low uncertainty categories. Case data from 34 CWD-positive farmed cervid operations in Minnesota and Wisconsin from 2002 to January 2019 were categorized by transmission risks exposure and evaluated for trends. The majority of case farms recorded high transmission risks (56%), which were likely sources of CWD, but many (44%) had only moderate or negligible transmission risks, including most of the herds (62%) detected since 2012. The presence of CWD-positive cervid farms with only moderate or low CWD transmission risks necessitates further investigation of these risks to inform effective control measures.

## 1. Introduction

CWD (chronic wasting disease) has emerged as one of the most important diseases of cervids due to its capacity for infectious spread, mortality in wild and farmed cervid populations, and associated economic damages to farmed cervid and hunting-related industries. The disease is a transmissible spongiform encephalopathy of cervids, in the same family as scrapie, bovine spongiform encephalopathy, and Creutzfeldt–Jakob disease [1,2]. The infectious agent of these diseases is widely accepted to be a misfolded form of the cellular prion protein, which is found naturally on the cell membranes of nervous system tissue [3]. The prion protein can also be expressed in other tissues throughout the body, including the heart, muscle, lymphoid tissues, kidneys, gastrointestinal tract, skin, and endothelium [3]. The function of this protein is currently not fully understood. Upon infection via ingestion or other route of entrance to a new host, PrP^CWD^ (infectious chronic wasting disease prion protein) travels up ascending fibers of the central nervous system, converting the body’s natural prion proteins into the infectious form [4]. After a variable incubation period of approximately 12–24 months, though longer in some animals, the result is an incurable progressive degenerative encephalopathy leading to death [1,2,4].

CWD was first discovered in mule deer (*Odocoileus hemionus*) in captive facilities in Colorado in the 1960s [5]. Since then, it has been detected in wild and farmed cervids in 26 U.S. states, Canada, South Korea, and recently in Norway, Finland, and Sweden [6,7,8,9]. The prevalence in certain free-ranging deer populations in North America, including areas of Colorado, Wyoming, and Wisconsin has reached as high as 30% [10,11]. A high prevalence of CWD may be detrimental to the sustainability of wild cervid populations [10,11]. Once established in these populations, current disease control practices have been largely ineffective in eliminating the disease [12,13].

CWD also poses a serious threat to farmed and captive cervids. In the upper Midwest U.S. states, including Minnesota and Wisconsin, the primary cervid species on cervid operations are white-tailed deer (*Odocoileus virginianus*) and elk (*Cervus canadensis*) [14]. The purposes of cervid operations, called ‘cervid farms’ in this study, are varied and include food production, trophy deer for hunt ranches, antler velvet and urine production, breeding for sale of offspring, pleasure (pets), and exhibition display [14]. The impact of CWD to cervid farms results from both the associated morbidity and mortality and the economic effects of control measures to limit spread of the disease. For farms with positive detections, control measures include either total herd depopulation or long-term quarantine, due to current lack of other available effective methods to eradicate the disease. Unaffected farms also must adopt CWD-related biosecurity improvements enforced through regulatory programs, including (in Minnesota and Wisconsin) 8-ft perimeter fencing and additional fencing requirements (which could include double perimeter fencing) in endemic areas to prevent contact with wild cervids [15,16].

A high level of scrutiny has been placed on cervid farms due to their potential as a source of CWD to wild and farmed cervid populations, especially from the potential spread of CWD over long distances through cervid movements. Currently, ELISA and IHC (immunohistochemistry) assays of the brain and/or lymph node are the only approved official CWD tests certified by USDA APHIS (United States Department of Agriculture Animal and Plant Health Inspection Service) and available to cervid farms, requiring postmortem tissue collection. These tests can be used antemortem on rectal associated mucosal lymphoid tissue, although the collection of this tissue can be expensive, distressing to the animals, and time-intensive [17,18]. In addition, USDA APHIS recognizes IHC as an official antemortem test only in certain circumstances, such as WTD (white-tailed deer) herds epidemiologically linked to CWD-positive herds, due to limitations of the test including decreased sensitivity for detecting CWD in certain host genotypes and in the early stages of the disease [18,19,20]. Thus, though there are extensive official postmortem sampling surveillance requirements, individual farmed cervids are typically untested prior to movement between herds, resulting in potential for undetected infected animals to spread CWD [21].

Despite the development of the U.S. CWD Voluntary Herd Certification Program, restricted movements of live cervids, and implementation of farm biosecurity practices and control measures to prevent CWD transmission (requirements varying by state), cases of CWD in farmed cervids continue to be detected. From 2017 to May 2019, there were 35 CWD-positive farmed cervid operations detected in the U.S.; 14 of these herds were enrolled and certified (having over 5 years of active surveillance among other requirements) in the U.S. CWD Voluntary Herd Certification Program [22]. In Minnesota and Wisconsin alone, adjacent states in the U.S. Great Lakes region with approximately 300 active cervid farms each, 34 farms were detected with CWD from the first case in 2002 to January 2019 [23,24]. There is substantial stakeholder concern about the continued spread of CWD among farmed cervids and potential spillover to wild cervid populations. This concern was further heightened due to a report not yet published in scientific literature of successful CWD transmission to rhesus macaques by consumption of CWD infected elk and deer meat or brain tissue, indicating potential health risks to humans [25].

While animal health regulatory officials have pursued detailed investigations into the CWD cases on these cervid farms, the long incubation period of the disease makes tracebacks and historical exposure assessments challenging. In addition, there is an incomplete understanding of CWD transmission and relevant risks to farmed cervids, such as environmental contamination. While CWD risk assessments have been conducted at country levels, farm-level risk assessments for individual cervid farms are not readily available [26]. Furthermore, populations of CWD-positive farms in the U.S. have not been systematically described in terms of transmission risks experienced, creating difficulty in determining best herd management practices and effective regulatory policy. To address these gaps, the objectives of this study were to:

1. Use current scientific understanding of CWD transmission and knowledge of farmed cervid operations to conduct a qualitative risk assessment for CWD transmission to cervid farms.
2. Applying this risk assessment, systematically describe the CWD transmission risks experienced by CWD-positive farmed cervid operations in Minnesota and Wisconsin.

## 2. Materials and Methods

### 2.1. Risk Assessment

The methodology for conducting a qualitative CWD transmission risk assessment was guided by OIE resources [27,28]. Emphasis was placed on entry and exposure assessment, as consequences were assumed to be equal for any CWD detection. A systematic literature search was conducted to assess possible means of transmission of CWD in farmed cervids. PubMed, CABI, Agricola, Zoological Record, Web of Sciences Core Collection, Scopus, and Wildlife and Ecology Studies were searched on 11 September, 2018. PubMed was searched twice using the mesh terms “Wasting Disease, Chronic” and “Wasting Disease, Chronic/Transmission.” CABI and Agricola were searched for “Chronic Wasting Disease” in the subject heading with AND “Transmission” in all fields. Zoological Record and Web of Sciences Core Collection were searched for “Chronic Wasting Disease” AND “transmission” in topic search. Scopus was searched for “Chronic Wasting Disease” AND ‘transmission’ in an article title, abstract, and keyword search. Wildlife and Ecology Studies Worldwide was searched for “’Chronic Wasting Disease” AND “transmission” with no fields selected. After manual deletion of duplicates, 884 articles of literature were identified.

For demonstrating a proven pathway of transmission, studies needed to meet the following criteria:

1. Demonstrate CWD transmission to cervids from specified sources in a controlled environment, which could be consistent with non-experimental settings (i.e., not intracerebral).
2. Demonstrate diagnostic confirmation of transmission with a diagnostic assay (including western blotting, immunohistochemistry, enzyme immunoassay, protein misfolding cyclic amplification assay, or real time quaking induced conversion assay).
3. Be published in English.

In addition to the systematic approach of assessing proven means of transmission, publications from the search results that identified potential yet not proven means of transmission to farmed cervids were also considered in our framework development. These included studies that showed means of prion environmental contamination (not necessarily CWD) or demonstration of prion or CWD infectivity by novel exposures to species other than cervids. The findings of certain studies published between the literature search period and submission of this study for publication deemed of significant consequence to CWD transmission and connected studies to provide context were also considered, and findings from this category were noted as “more recent studies” when included.

### 2.2. Case Description

Investigative data of the 8 Minnesota and 26 Wisconsin farms with CWD detected in cervids on their premises from 2002 to January 2019 were obtained through cooperation with the Minnesota Board of Animal Health and the Wisconsin Department of Agriculture, Trade and Consumer Protection. In addition, supplemental information relevant to our study and not included in the written records (due to variability in investigation techniques and queries, especially in older cases, and inadvertent loss of records including due to fire) was obtained from personal correspondence with active personnel at both agencies. Final study categorizations and conclusions of the cases were also reviewed for accuracy by these personnel.

Using the transmission risk assessment, each CWD-positive farm was categorized by transmission risks experienced based on available information. Patterns of these categorizations in the case data set were examined by year, state, and proximity to CWD positive wild populations. In addition, CWD surveillance data was compiled from the 5 years before index case detection for each case farm since 2014, when standardized investigative worksheets were available. The five years of surveillance data was chosen due to the record-keeping requirements of 5 years in Wisconsin [29].

## 3. Results

### 3.1. Proven Pathways of PrP^CWD^ Transmission

Thirty-four studies demonstrated CWD transmission in controlled settings (Table 1). Of these, 27 studies demonstrated experimental CWD transmission utilizing CWD-positive inocula via oral, intranasal, intravenous (IV), or intraperitoneal (IP) routes. Four studies showed transmission through direct contact with infected cervids, with oral or intranasal exposure the most likely means of transmission. Five studies showed transmission through indirect contact with infected cervids by shared environments or fomites. Two studies demonstrated in-utero CWD transmission. These proven means of transmission indicated that both direct and indirect contact pathways needed to be considered for the risk assessment.

### 3.2. Infectious Dose of PrP^CWD^

The minimum infectious dose of CWD prions needed for transmission to occur by each possible transmission route and the effects of dose on the time course of disease have not been definitively determined. Oral transmission to deer has been achieved with an estimated 3 µg of PrP^CWD^ from single doses of 0.5 or 1 g of CWD positive brain, though more recent studies show required doses may be lower as transmission occurred from a single dose of 10 mg CWD positive brain and from as little as 300 ng total of CWD positive brain administered as three 100 ng weekly doses [34,64]. Other studies have demonstrated that lower doses may be possible for intranasal transmission [52,53]. Most studies, however, were conducted as proof of concept or to examine another objective and utilized expected high doses of PrP^CWD^ to ensure transmission. The minimum dose of PrP^CWD^ required for transmission could vary by frequency of exposure, species, genotype, exposure route, and exposure substance. Thus, any potential pathway of entry and exposure for PrP^CWD^ was not ruled out in the assessment. Many pathways, however, had a high level of uncertainty as they could have involved low dose exposures and lacked proven experimental transmission.

### 3.3. Sources of PrP^CWD^

#### 3.3.1. Tissue Sources

Lymphoid, brain, and blood were the only body tissues experimentally proven capable of transmitting CWD to cervids (Table 1). Brain and lymphoid material have been identified as tissues with the highest concentration of PrP^CWD^ in infected animals [4,33]. As little as 0.01 g solid brain tissue PO or 5 mg solid brain tissue aerosolized intranasally has been shown to be infectious [30,34,52]. Tonsillar tissue was infectious with a 1 mL of 10% wt/vol suspension PO [40]. Large volumes of blood (150–250 mL) were the vehicle of experimental transmissions by IP or IV injection, with PrP^CWD^ likely harbored in B cells and platelets [49,51,54,56].

PrP^CWD^ has also been detected in CWD-positive cervids in other tissues including the pancreas, adrenal gland, fat, cardiac muscle, and antler velvet [33,43,65,66,67]. Henderson et al. (2015) estimated the LD_50_ equivalent concentration of PrP^CWD^ in the left ventricle, pancreas, jejunum, and spleen to be 50 times lower than the obex [33]. Exact concentrations of PrP^CWD^ within lower concentration tissues and implications for infectivity have not been fully determined. These tissues, however, may play significant roles in environmental contamination from carcasses when considering ratios of body mass of whole carcass to brain or annual shedding of antler velvet.

#### 3.3.2. Secreta and Excreta Sources

Saliva and combined urine and feces were the only secreta and excreta experimentally proven capable of transmitting CWD to cervids (Table 1). Transmission from saliva was demonstrated experimentally with oral doses of 50 mL of saliva split over three or five days [49,51]. Oral doses of 50 g of feces and 50 mL urine divided over 3–14 days or 85 mL of urine and 112.5 g of feces divided over 90 days resulted in evidence of infection in WTD [47,49,51]. Studies have detected PrP^CWD^ in saliva, feces, and urine of infected cervids as soon as 3 months, 14 days, and 3 months respectively post-inoculation [33,36,68,69,70]. Henderson et al. (2015) estimated that the quantity of urine needed to contain 1 LD50 of PrP^CWD^ was 10–20 mL, compared to 5–10 mL of saliva, an indication that saliva likely plays a major role in direct contact transmission [33]. The discharge of PrP^CWD^ in urine, feces, and saliva over extended periods of time likely also plays significant roles in environmental contamination and spread of CWD. More recent studies also have detected PrP^CWD^ in WTD male reproductive tissues and fluid, though this transmission potential has not been fully explored [71]. A previous study attempting scrapie transmission to rodent models using intracerebral injections of semen from infected rams at varying stages of the disease was not successful [72].

#### 3.3.3. Environmental Sources

PrP^CWD^ has been shown to contaminate and persist in the environment. Miller et al. (2004) demonstrated transmission to cervids in environments contaminated with infected deer carcasses or live cervids 1.8 years and 2.2 years earlier [62]. Four other laboratory studies also demonstrated cervid CWD transmission with continued indirect contact between cervids, implying environmental or fomite contamination [49,57,60,61]. Transmissible spongiform encephalopathy prions, including PrP^CWD^, have been found to bind to soil, with some clay or soil types potentially increasing infectivity of the prions [53,73]. Aside from binding to soil, other prions have been shown to maintain infectivity after binding or adhering to aluminum, brass, cement, polypropylene, stainless steel, wood, and mineral licks [74,75]. PrP^CWD^ has also been shown to be uptaken and bound in plants, and, though not tested with PrP^CWD^ in cervids, other strains of prions have been shown to maintain infectivity after plant binding [76]. Low levels of PrP^CWD^ have been detected in environmental water samples in CWD endemic areas [77]. In addition, uninfected and non-susceptible animals (coyotes, crows) may serve as potential spreaders of contamination, as studies show retained infectiousness after infectious PrP (CWD or scrapie) passage through gastrointestinal tracts [78,79].

### 3.4. CWD Entry and Exposure Risk Categorization

#### 3.4.1. Tissue Transmission Risk

The presence of prions in most body tissues, proven ability of carcasses to contaminate environments, and infectious dose uncertainty indicated a high level of transmission risk for any direct contacts with tissue from infected cervids. Whole tissue entry to farms may occur from hunting or taxidermy practices bringing wild cervid parts onto farm premises, with uncertainty in transmission risk related to the tissue CWD status. The highest entry risk in this circumstance was indicated for tissues from areas with CWD-positive wild cervids, with lower risk but higher uncertainty indicated for tissues from other or unknown status areas. Without detailed tissue handling or disposal practices to prevent PrP^CWD^ premise contamination, the exposure risk once these tissues are brought on farm may be substantial. In addition, though experiments used large quantities of blood to achieve IP or IV transmission (not realistic in a non-experimental setting), transmission risk from gross blood and other tissue entry and exposure from procedure equipment sharing between farms, while unlikely, remained a possibility.

#### 3.4.2. Secreta and Excreta Transmission Risk

Demonstrated prion presence and infectivity of secreta and excreta indicated transmission risk from their ingestion or other direct exposure. This transmission pathway likely involves direct contact with infectious farmed or wild cervids or invasive reproductive procedures.

Farmed cervid entry and exposure risk, while having the possibility of occurring through adjacent neighboring farms, was determined to most likely occur from cervid movements between operations. Movements from farms with positive cervids indicated the highest risk of CWD transmission. Of note, no cervid shipments from farms after detection of CWD-positive cervids on these farms to farms without detected CWD have been legally allowed since CWD was first detected in the farmed cervid populations in Minnesota and Wisconsin [16,80]. Instead, exporting cervid farms were typically retroactively discovered to be CWD-positive through postmortem surveillance. Cervid introductions from farms under routine postmortem surveillance with no CWD-positive test results indicated non-negligible level of risk with high uncertainty.

Direct contact transmission risk from wild cervids could result from wild cervid introductions, fence breaches resulting in farmed cervid escape, interactions with wild cervids, and reentry, or nose-to-nose contacts through the fence itself in areas near CWD-positive wild cervids. Entry and exposure risks were classified as high for farms with wild cervid introductions within perimeter fences or farmed cervid escapes and reentry after fence breaches. To assess the risk posed by direct contact with wild cervids through farm perimeter fencing, VerCauteren et al. (2007) found that elk had limited contact through single fencing with wild elk, but no contact with wild mule deer [81]. In contrast, in another study, farmed WTD had little direct contact with wild WTD through fencing [82]. No contacts were observed through double fencing [81]. Based on these studies, double perimeter fencing indicated a negligible risk of transmission, while single perimeter fencing indicated a non-negligible level of risk but with a high level of uncertainty.

Finally, though the recent evidence of PrP^CWD^ presence in reproductive tissues and fluids suggested that invasive advanced reproductive techniques could provide a route of sexual transmission, without proven CWD transmission from this pathway, much uncertainty exists.

#### 3.4.3. Environmental Transmission Risk

Due to proven transmission studies, indirect contact pathways of entry from sharing equipment, feed, or other materials from a known CWD-positive farm indicated a high risk for transmission, with the assumption of exposure of the material to cervids after entry to premises. Without further evidence for proven transmission, however, estimation of entry and exposures risks for other environmental and fomite sources of CWD indicated a non-negligible level of risk with a high level of uncertainty. Many cervid operations are located in areas near CWD-positive wild cervids, and the level of contamination of these natural environments and their infectious potential remained largely unexplored.

#### 3.4.4. Risk Categorization

Based on the literature review, our risk assessment criteria stratified combined entry and exposure risks into three overall transmission risk levels for indirect contact and direct contact categories; high risk with low uncertainty, moderate risk with high uncertainty, and negligible risk with low uncertainty (Table 2). Risk uncertainty from current evidence meant that moderate risks included a wide range between the extremes of high risk to negligible risk.

For applicability to the case farms, proximity to CWD-positive wild cervids was classified as a risk for CWD transmission only if CWD had previously been detected in the surrounding wild population (<80 km from the operation) and within 5 years of detection of CWD on the farm (including after initial farm CWD detection). The 80 km cutoff was used as a conservative home-range distance reported in official investigation reports for the cases considered in this study. Other reports have demonstrated that free-ranging deer in the region can have movements up to 112 km, though average deer movements are much shorter [83,84]. The 5-year delay was used to account for highly variable sampling of wild cervid populations depending on location within Minnesota and Wisconsin with subsequent potential for delayed detection in wild cervid populations [85,86]. Cervid movements and wild cervid contacts were considered to increase CWD risk to farms if they occurred within the previous 5 years before detection. Five years was chosen due to current CWD regulations which set a 5-year quarantine period for suspect CWD-exposed herds and 5-year record keeping requirements [20,29].

### 3.5. Case Farms Description

**Figure 1:**
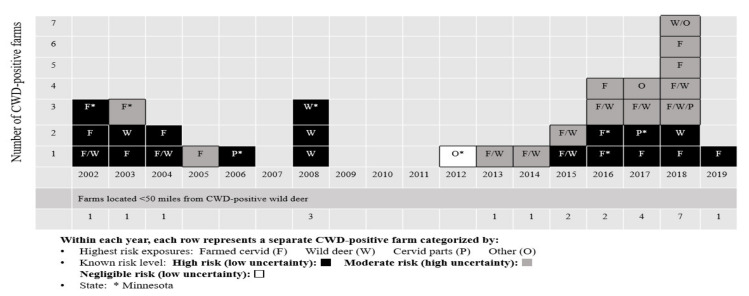
Highest transmission risks by year for Minnesota and Wisconsin CWD-positive cervid farms.

The risk assessment criteria were used to classify the 34 CWD-infected cervid farms in Minnesota and Wisconsin by transmission risks (Table 3, Figure 1). Case farms were detected from 2002 to January 2019, with a total of eight cases in Minnesota and 26 cases in Wisconsin. There was a period of three years between detection of an earlier group of farms from 2002 to 2008 and a later group from 2012 to 2019, with no regulatory or epidemiologic explanation found for the gap. Twenty-one of the CWD-positive farms (62%) tested positive from 2012 to January 2019, indicating a trend toward increasing numbers of positive farms in recent years. Six of the farms were comprised of exclusively elk at detection, 23 contained exclusively WTD, and five contained a mixture of elk, WTD, and/or other species. Twenty-three of the cervid operations were exclusively breeding farms, and nine were either hunting preserves (where hunters paid to hunt farmed deer within perimeter fencing) or joint hunting preserves and breeding farms. Two farms had deer for other primary purposes.

All case herds were in compliance with state testing standards at the time of index case detection. Surveillance data on the total number of negative CWD tests in the previous 5 years before index case detection and the herd size at index case detection for the CWD-positive farms from 2014 to January 2019 was consistently recorded (Table 3). Other numerical data indicative of herd CWD surveillance including yearly deaths and test results of trace-out animals were either not consistently available from case investigation records or not reported.

### 3.6. Categorization of CWD-Positive Farms by Transmission Pathway and Risk Level

#### 3.6.1. Direct Contact with Infected Farmed Cervids

Overall, 29 (85%) of the CWD-positive farms reported direct contact with other farmed cervids as a transmission risk. Twelve farms (35%) experienced a high transmission risk from introducing cervids from farms later found to be CWD-positive. In some of these cases, cervids were traded at the same time or exchanged between test-positive farms over a period of years, and the true source farm was unable to be determined. Six of the 21 CWD-positive farms (29%) detected since 2012 had this high transmission risk, compared to 6 out of the 13 (46%) farms detected in the earlier cluster. Seventeen of the CWD-positive farms (50%) experienced a moderate transmission risk from introducing at least one cervid in the previous five years from farms without positive CWD-positive tests (none of these source farms had reported a positive CWD test as of June 2020). The remaining 5 CWD-positive farms (15%) reported being closed to introductions of new cervids for at least the previous five years.

#### 3.6.2. Direct Contact with Infected Wild Cervids

Overall, 22 of the CWD-positive farms (65%) experienced transmission risks from direct contact with wild cervids. Eight farms (24%) experienced a high transmission risk from farmed cervid escape and re-entry to the premises or wild cervid entrance. This type of high transmission risk was experienced by 2 of the 21 CWD-positive farms (10%) detected since 2012, compared to 6 of the 13 (46%) farms detected previously. Fourteen (41%) of the CWD-positive farms experienced a moderate transmission risk from being located in areas near CWD-positive wild cervids and having single perimeter fencing. Of the remaining 12 CWD-positive farms (35%), nine were not located near a detected CWD-positive wild cervid (77%), and the other three were located near CWD-positive wild cervids but had double perimeter fencing (23%) for at least the previous 5 years.

#### 3.6.3. Indirect Contact with Cervid Tissues from Hunting or Taxidermy

Overall, three of the CWD-positive farms (9%) experienced exposure to cervid parts through hunting or taxidermy practices. Two farms experienced a high transmission risk from reported cervid part introduction to the cervid housing areas of the premises from known wild cervid CWD-positive areas. Another farm experienced a moderate transmission risk from introducing cervid parts to the premises from unknown locations and exposed farmed cervids to areas where the parts were processed. The remaining 31 farms (91%) did not introduce cervid parts from outside deer onto their farms.

#### 3.6.4. Indirect Contact through Other Transmission Risks

Information related to other potential farm CWD exposures was only sporadically collected or reported, especially during earlier years of case investigations (also not included in Table 3). One farm (Farm 26, Table 3) in a CWD-positive area reported usage of feedstuff grown in a nearby field heavily exposed to wild cervids prior to being fed to farmed cervids. Another farm (Farm 28, Table 3), again located near CWD-positive wild cervids, reported significant washout of soil from heavy rainfall from outside the perimeter fencing into the deer pens and drinking ponds over a period of years, and had observed heavily used wild deer trails and activity near the fencing.

#### 3.6.5. Aggregate Farm Risk

When considering all potential CWD transmission risks, 19 farms (56%) experienced at least one high risk, 14 (41%) experienced at least one moderate/uncertain risk, and one farm (3%) experienced only negligible risks. Of the eight Minnesota and 26 Wisconsin CWD-positive farms, respectively, 6 (75%) and 13 (50%) experienced at least one high risk, 1 (13%) and 13 (50%) experienced at least one moderate/uncertain risk, and one (13%) and zero farms experienced only negligible risks. Eleven of the 15 farms (73%) without high transmission risks were located within 80 km of a CWD-positive wild cervid. Twelve of these 15 farms (80%) added cervids from herds with no history of CWD detections in the previous 5 years, while three (20%) had no additions during that time. Eleven of the 13 herds (84%) detected prior to 2012 experienced at least one high transmission risk, compared to 8 of the 21 herds (38%) detected since 2012. Of the 13 herds without high known risk exposures detected since 2012, 10 (77%) reported animal additions within the previous 5 years before detection, and 11 (73%) were located within 80 km of a CWD-positive wild cervid; eight (73%) had single perimeter fencing and three (27%) had double perimeter fencing.

## 4. Discussion

This investigation was conducted to improve understanding of CWD transmission risks to cervid farms, as CWD-positive farms continue to be detected despite existing herd disease prevention programs and regulatory measures. To our knowledge, this is the first study to conduct a qualitative CWD transmission risk assessment for cervid farms and systematically describe transmission risks experienced by a population of CWD-positive farms.

The majority of CWD-positive cervid farms in Minnesota and Wisconsin (19/34, 56%) experienced at least one high transmission risk (introducing cervids from another farm later detected with CWD (12/19, 63%), having reported wild deer enter their farm pens or farmed animals escape and re-enter in areas with CWD in the wild deer population (8/19, 42%), or entrance of high risk cervid parts through hunting or taxidermy practices (2/19, 11%)). For farms which experienced more than one high transmission risks, it was not possible to identify which of these risks led to CWD infection. Risks of CWD introduction through cervid movement from a CWD-positive farm or through direct contacts with wild cervids in CWD endemic regions are generally well understood and targeted in herd disease control programs and regulatory policy. One potential primary cause of these direct contact transmission risks from farmed cervids was the low sensitivity of the current CWD surveillance system, with contributing factors including postmortem testing of animals with varying numbers of animals available annually and imperfect test sensitivity at earlier stages of the disease. In addition, cervid farmers may not have fully recognized the limitations of current postmortem CWD surveillance and the risk from purchasing animals from herds under surveillance with no history of CWD-positive tests. Among other possible improvements, certification of more sensitive testing strategies using new technologies, such as RT-QuIC (real-time quaking induced conversion) or sPMCA (serial protein misfolding cyclic amplification) in official disease management programs may be an important step to improving CWD surveillance and preventing CWD transmission, as they allow detection of CWD at lower concentrations in samples, earlier in the disease course, and may be used on more easily accessible tissue and excreta samples for antemortem testing [33,70].

Regarding other high transmission risks, some cervid producers may not have fully understood the infectious risk potential from wild cervids and cervid parts, especially in areas with CWD-positive wild cervids. Cases with reported high risk direct wild cervid contacts were caused by various situations, including gate and fencing breaches, resulting in potential exposures to wild cervids. While this high risk occurred in nearly a quarter of the cases overall, it was less common in recent cases; 2/21 (10%) of the CWD-positive farms detected since 2012 experienced this risk compared to 6/13 (46%) of the farms detected previously. Farmer biosecurity improvements and increased regulation and enforcement may have driven this change. In terms of entry of wild cervid parts to farms, education to hunters and cervid farmers regarding carcass and part contamination and difficulties of proper PrP^CWD^ decontamination may have minimized this risk through time.

Notably, 15/34 (44%) of CWD-positive farms did not experience high CWD transmission risks, indicating the potential importance of moderate transmission risks (with associated high uncertainty). Moreover, 12/15 (80%) of these herds had recent herd additions from farms with no CWD-positive tests, which may have been a source of entrance (though there have been no detections in the source farms). While unreported movement of animals was possible, this was not considered a substantial factor in these cases due to careful case investigation and review of both herd records and prior inspection data. Eleven of the 15 (73%) CWD-positive farms without high-risk exposures, however, were located within 80 km of known CWD-positive wild deer. Single perimeter fencing was present on 8/11 (73%) of these farms which may have allowed direct contact between farmed and wild cervids through intact fencing, though this risk has been shown to be low, especially for WTD [81,82]. Single perimeter fencing, however, is more vulnerable than double perimeter fencing to breaches which allow the ingress and egress of cervids. Furthermore, the fence itself could potentially serve as a fomite after contact with a wild cervid [75].

CWD was detected despite double perimeter fencing (with no reporting of fencing breaches) on 3/11 (27%) of the farms without higher known risk exposures in areas with CWD-positive wild cervids. CWD detection in these herds, with no known recent cervid movements from other CWD-positive farms, indicates the potential significance of indirect contact exposures in locations with infected wild cervids. In one case, the primary CWD transmission risk from a double-fenced herd in an endemic area was potentially contaminated feed. Another farm (though single fenced) in a CWD endemic area had reported potential water and soil contamination. For cervid farms located in endemic CWD areas, these indirect contact pathways may serve as a primary means of transmission.

This risk assessment identified much uncertainty regarding the potential for environmental sources leading to CWD transmission. Large gaps in understanding of CWD transmission from soil, plants, other fomite sources, water, and scavenger and predator feces to cervids remain. This is cause for concern when considering the findings from this case series analysis and the limited options available to effectively manage transmission risk from these sources. Use of recently developed PrP^CWD^ detection techniques, such as RT-QuIC and sPCMA to detect prions in environmental sources at lower concentrations than previously possible provide an opportunity to investigate risks from these indirect exposure routes [33,87]. Specifically, studies could quantify PrP^CWD^ contamination and infectious potential of water and commonly used feeds in CWD-endemic areas and on case farms. In addition to those knowledge gaps, information on indirect contact transmission risks to farms was often not available from case investigations. A more detailed investigation of recently detected CWD-positive farms is warranted to assess these indirect contact risks, along with collection of data from non-positive farms, of which little is currently known regarding practices relevant to these risks.

The case analysis identified changing trends of CWD transmission risks over time in CWD-positive herds. Despite a numerical increase in CWD-positive farm detection, only 8/21 (38%) of the recently detected farms experienced high transmission risks, compared to 11/13 (85%) of the earlier cases. Several possibilities could explain this trend. Regulatory policies implemented over time targeting known high transmission risk exposures may have been effective; similarly, changing management practices on farms may have reduced these transmission risks. Conversely, a buildup of prion contamination in the environment may have occurred in CWD endemic areas during the study period, where 11/13 (85%) of recent cases without high transmission risks were located, and increased environmental or other means of transmission.

Seventeen of the 21 (81%) recently detected farms since 2012 were located in Wisconsin. Though the number of cervid farms in each state is similar, differences in regulations and in CWD prevalence and distribution in free-ranging cervids may have contributed to this observation. CWD has been detected in free-ranging WTD in at least 33 of 72 Wisconsin counties spread across the state [85,88]. In comparison, Minnesota’s free-ranging WTD endemic zones only include 9 out of 87 counties to date and are primarily confined to the southeast corner of the state [89]. Sixteen of the 17 (94%) CWD-positive farms in Wisconsin since 2012 have been located within 80 km of known CWD-positive wild deer, supporting the potential for both direct and indirect contacts associated with wildlife leading to transmission risks, especially in these regions with endemic CWD in wild populations.

This case series investigation had some limitations. Risk-based surveillance for CWD in the wild cervid population varied by state and county over the course of this dataset, with the likelihood of delayed findings of farm proximity to CWD-positive wild cervids until after CWD detection in a farmed cervid population. In addition, due to the latent period of CWD infection, index case detection on CWD-positive farms may have substantially lagged behind the actual date of first CWD transmission to the farm, and the index case detected may not have been the initial infected animal. It is also possible (though unlikely) that other unreported transmission risks such as cervid introductions which occurred more than 5 years prior to detection may have been the source of CWD rather than reported transmission risks within that time frame.

Surveillance systems are in place with the objective of timely detection of CWD in farms but are different in Minnesota and Wisconsin. Minnesota currently requires all farmed cervids over 12 months of age that are slaughtered, killed, or die on the premises to be tested for CWD [90]. In contrast, Wisconsin requires testing (at minimum) of cervids at least 16 months of age in the following categories: 100% that die by accident or natural cause, 50% that are killed by hunting on hunting preserves, 50% that are killed intentionally, and 25% that are shipped to a slaughter plant [91]. In Wisconsin, herds planning to move live cervids are required to test higher percentages and younger animals [91].

Surveillance data from CWD-positive herds detected since 2014 showed that all of these herds had histories of cervid surveillance (Table 3), and none were reported out of compliance. Continuous surveillance of the CWD-positive farms provides evidence against long histories of infection in the herds before index case detection, despite less than 100% testing on some farms. Currently approved CWD tests (IHC and ELISA) can detect subclinically infected cervids and, therefore, the potential for the biased sampling of healthy appearing cervids to obscure CWD-positive herd status over long periods is unlikely to be substantial [43]. Prevalence at depopulation was not useful in inferring length of infection on farm, since multiple management factors such as pen size, animal density, frequency of contacts, and on-farm animal movements in addition to the method of introduction could have also had significant effects on CWD herd prevalence. Some farms also had substantial delays before they were depopulated after index case detection.

Another limitation came from investigative case records varying in quality between farms, states, and years, which impeded the ability to fully assess and compare transmission risks. Through time, the questions asked and observations recorded changed with increasing knowledge about CWD. Much of the information regarding wild cervid contacts and potential indirect exposures was obtained from records of interviews with the producers and subject to errors due to recall bias and other factors. It should be noted, however, that investigations were not solely based on owner interviews but paired with prior knowledge of the farm from regulatory inspections, communications, and official animal movement records. Nonetheless, a more detailed standardized investigatory protocol for use across states would allow for improved risk assessments on farms.

Differences between states and counties could have influenced the categorization of the CWD-positive farms into the risk categories in this study. Due to the overall small number of CWD-positive farms and data availability limitations, certain potential confounders could not be further explored in this case series study. This includes the proximity to CWD-positive wild deer, a categorization which can be affected by differences in county wild cervid CWD prevalence and surveillance intensity. Future evaluations should address these CWD transmission risks to farmed cervids using a case-control protocol to allow comparisons between CWD-positive and CWD-negative farms.

## 5. Conclusions

This qualitative CWD risk assessment identified several moderate transmission risks with associated high uncertainty along with the well-understood high and negligible transmission risks. For the CWD-positive farms detected in Minnesota and Wisconsin, high transmission risks were the likely source of CWD in the majority of cases, but cervids on many other farms (including a higher proportion of recent cases) likely acquired CWD through moderate transmission risks. This category of moderate transmission risks presents opportunities for further research to provide the scientific basis to inform improved CWD mitigation strategies. Additionally, for more robust surveillance and monitoring systems, industry stakeholders should be encouraged to adopt recently developed higher sensitivity diagnostic techniques, such as RT-QuIC and sPCMA.

## Figures and Tables

**Table 1:** Studies demonstrating CWD transmission in controlled settings.

Mode of Transmission	Species	Infectious Material or Animals
Inoculation:		
Oral		
1. Davenport 2017 [30], Hoover 2017 [31], Goni 2015 [32], Henderson 2015 [33], Miller 2012 [34], Johnson 2011 [35]	WTD	CWD + WTD Brain
2. Plummer 2017 [36], Gordon 2009 [37], Hamir 2006 [38]	Elk	CWD + Elk Brain
3. Nalls 2013 [39]	Reeves’s Muntjac Deer	CWD + WTD Brain
4. Wolfe 2012 [40]	Mule Deer	CWD + Mule Deer Tonsil
5. Basu 2012 [41]	Rocky Mountain Elk	CWD + Elk Brain
6. Mitchell 2012 [42]	Reindeer	CWD + WTD Brain
7. Miller 2012 [34], Fox 2006 [43], Sigurdson 1999 [44]	Mule Deer	CWD + Mule Deer Brain
8. Pushie 2011 [45]	Elk	CWD + Brain (sp. unspecified)
9. Balachandran 2010 [46]	Red Deer	CWD + Rocky Mountain Elk Brain
10. Haley 2009 [47]	WTD	CWD + Mule Deer Urine + Feces
11. Martin 2009 [48]	Red Deer	CWD + Elk Brain
12. Mathiason 2009 [49]	WTD	CWD + Deer sp. Brain
13. Mathiason 2009 [49]	WTD	CWD + Deer sp. Saliva
14. Kreeger 2006 [50]	Shiras Moose	CWD + Mule Deer Brain
15. Mathiason 2006 [51]	WTD	CWD + Mule Deer Saliva
16. Mathiason 2006 [51], Mathiason 2009 [49]	WTD	CWD + Mule Deer Brain
Intranasal		
1. Denkers 2013 [52], Nichols 2013 [53]	WTD	CWD + WTD Brain
Intraperitoneal		
1. Davenport 2018 [54]	WTD	CWD + WTD Blood
2. Mathiason 2009 [49]	WTD	CWD + Mule Deer Blood
Intravenous		
1. Angers 2014 [55]	WTD	CWD + Transgenic Mice Brain
2. Mathiason 2010 [56]	WTD	CWD + WTD Blood
3. Mathiason 2009 [49]	WTD	CWD + Deer sp. Blood
4. Mathiason 2009 [49], Mathiason 2006 [51]	WTD	CWD+ Mule Deer Blood
Direct Contact:		
1. Davenport 2018 [54]	WTD	CWD + WTD
2. Moore 2016 [57]	Reindeer	CWD + Reindeer
3. Rhyan 2011 [58], Miller 2003 [59]	Mule Deer	CWD + Mule Deer
Indirect Contact:		
1. Moore 2016 [57]	Reindeer	CWD + Reindeer, Adjacent Pen No Direct Contact
2. Wolfe 2014 [60], Pilon 2013 [61]	Mule Deer	CWD + Mule Deer, Same Pen
3. Mathiason 2009 [49]	WTD	CWD + WTD Feed Buckets, Water, Bedding
4. Miller 2004 [62]	Mule Deer	CWD + Mule Deer Carcass, Same Pen
5. Miller 2004 [62]	Mule Deer	CWD + Mule Deer, Same Pen
In Utero:		
1. Selariu 2015 [63]	Rocky Mountain Elk Fetus	CWD + Rocky Mountain Elk
2. Nalls 2013 [39]	Reeves’s Muntjac Deer, Fetus	CWD + Muntjac Deer

**Table 2:** Risk of CWD transmission exposures to cervid farms.

Transmission Pathways	High Risk (Low Uncertainty)	Moderate Risk (High Uncertainty)	Negligible Risk (Low Uncertainty)
Direct contacts with infected cervids			
Introduction of farmed cervids	From farm later found to be CWD-positive	From farms with no CWD test positive animals in the 5 years before detection	No record of introductions from other farms in the 5 years before detection
Contact with wild cervids from farm location <80 km from a CWD-positive wild cervid detection	Farm cervid escapes/re-entry or wild cervid entry	Single perimeter fencing	Double perimeter fencing or not <80 km from a wild CWD detection
Indirect contacts with infected cervids			
Introduction of cervid parts (hunting, taxidermy)	From <80 km from CWD-positive wild cervids	From other areas	No introductions
Introduction of contaminated equipment, feed, water, or other fomites, scavenger entrance	From CWD-positive farms	From location <80 km from CWD-positive wild cervids or with farms with no CWD test positives	No indirect contacts

**Table 3:** Exposure risks of Minnesota and Wisconsin CWD case farms, 2002–2019.

			Exposure to Farmed Cervids		Exposure to Wild Cervids		Exposure to Cervid Parts	Surveillance	
Farm	Year of Detection	State	From CWD Positive Farm	Other Herd Additions ≤5 Years	Located ≤80 km from wild CWD Detection and Direct Contact	Located ≤80 km from wild CWD Detection (Single or Double Fence)	From CWD Positive Area (P) or CWD Status Unknown Area (U) (Hunting or Taxidermy)	Number of CWD Tests in Previous 5 Years	Herd Size at Index Case Detection
1	2002	MN	X	X					
2	2002	WI	X	X					
3	2002	WI	X	X	X	X (SF)			
4	2003	MN		X					
5	2003	WI	X	X		X (SF)			
6	2003	WI		X	X	X (SF)			
7	2004	WI	X	X					
8	2004	WI	X	X	X	X (SF)			
9	2005	WI		X					
10	2006	MN					P (H)		
11	2008	MN			X	X (SF)			
12	2008	WI		X	X	X (SF)			
13	2008	WI		X	X	X (SF)			
14	2012	MN							
15	2013	WI		X		X (SF)			
16	2014	WI		X		X (SF)		14	51
17	2015	WI	X	X	X	X (SF)		28	281
18	2015	WI		X		X (SF)		129	450*
19	2016	MN	X	X		X (SF)		153	143
20	2016	MN	X	X				56	15
21	2016	WI		X		X (SF)		12	17
22	2016	WI		X				1634	2080
23	2017	MN		X		X (SF)	P (T)	16	8
24	2017	WI		X		X (SF)		92	292
25	2017	WI	X	X		X (SF)		201	178
26	2017	WI				X (DF)		18	107
27	2018	WI		X		X (SF)	U (T)	27	95
28	2018	WI				X (SF)		10	10
29	2018	WI		X		X (DF)		12	6
30	2018	WI		X		X (SF)		145	274
31	2018	WI		X	X	X (SF)		9	15
32	2018	WI	X	X		X (SF)		389*	183
33	2018	WI		X		X (DF)		5	33
34	2019	WI	X	X		X (SF)		190	140
Total (RL: N, %) **			HR: 12, 35%		HR: 8, 24%		HR: 2, 6%		
			MR: 17, 50%		MR: 14, 41%		MR: 1, 3%		
			NR: 5, 15%		LR: 12, 35%		LR: 31, 91%		

* Best estimate based on case investigation records. ** RL = risk level by risk pathway (as detailed in Table 2) for either the introduction of farmed cervids (exposure to farmed cervids), contact with wild cervids from farm location <80 km from a CWD-positive wild cervid (exposure to wild cervids), or the introduction of cervid parts (hunting, taxidermy) (exposure to cervid parts). HR = high risk, low uncertainty, MR = moderate risk, high uncertainty, NR = negligible risk, low uncertainty.

## Data Availability

Not applicable.

## Abbreviations

CWD	Chronic wasting disease
PrP^CWD^	Infectious chronic wasting disease prion protein
WTD	White-tailed deer
IHC	Immunohistochemistry
RT-QuIC	Real-time quaking induced conversion
sPMCA	Serial protein misfolding cyclic amplification
USDA	United States Department of Agriculture Animal and Plant Health
APHIS	Inspection Service
